# TNFSF14/LIGHT, a Non-Canonical NF-κB Stimulus, Induces the HIF Pathway

**DOI:** 10.3390/cells7080102

**Published:** 2018-08-08

**Authors:** Laura D’Ignazio, Michael Batie, Sonia Rocha

**Affiliations:** 1 Center for Gene Regulation and Expression, School of Life Sciences, University of Dundee, Dundee DD15EH, UK; l.dignazio@dundee.ac.uk; 2 Department of Biochemistry, Institute of Integrative Biology, University of Liverpool, Liverpool L697ZB, UK; m.batie@liverpool.ac.uk

**Keywords:** HIF, NF-κB, EPAS1, LIGHT, TNFSF14, p52, NIK, ChIP

## Abstract

Non-canonical NF-κB signalling plays important roles in the development and function of the immune system but it also is deregulated in a number of inflammatory diseases. Although, NF-κB and HIF crosstalk has been documented, this has only been described following canonical NF-κB stimulation, involving RelA/p50 and the HIF-1 dimer. Here, we report that the non-canonical inducer TNFSF14/LIGHT leads to HIF induction and activation in cancer cells. We demonstrate that only HIF-2α is induced at the transcriptional level following non-canonical NF-κB activation, via a mechanism that is dependent on the p52 subunit. Furthermore, we demonstrate that p52 can bind to the HIF-2α promoter in cells. These results indicate that non-canonical NF-κB can lead to HIF signalling implicating HIF-2α as one of the downstream effectors of this pathway in cells.

## 1. Introduction

NF-κB is the name of a family of transcription factors that is mostly known for their function in regulation of the cellular response to infection and inflammation [1], but it also has pleotropic functions in responses to a number of cellular stresses, such as DNA damage and hypoxia [2]. Given its role in the control of inflammatory responses, it has also been widely associated with cancer, where it can act as both as tumour suppressor and tumour promoter [3,4].

NF-κB encompasses five genes that give rise to seven proteins, RelA/p65, RelB, cRel, NFKB1/p105 and p50, and NFKB2/p100 and p52 [5]. Due to its importance, it is kept under several strict control mechanisms, such cellular localisation, binding to inhibitor proteins and numerous negative feedback loops [6]. Activation of NF-κB follows, in a simplified view, three main pathways: canonical, non-canonical, and atypical activation pathways, which are dependent on the involvement of the upstream kinases called Inhibitor of κB (IκB) kinases (IKK) [2]. The canonical pathway, responding to stimuli, such as TNF-α and IL-1β, activates the IKK complex, leading to the phosphorylation and inactivation of IκB proteins, and the consequent release of the NF-κB dimers, mainly containing RelA and p50 [5]. Once translocated into the nucleus, NF-κB subunits can receive additional control mechanisms, such as post-translational modifications [7], to induce or repress the specific and appropriate complement of target genes. In the non-canonical pathway, stimuli, such as B-cell activating Factor, or Lymphotoxin-β, activate the NF-κB activating Kinase (NIK), which phosphorylates and activates IKKα, leading, in turn, to the phosphorylation and processing of p100 into p52 [8]. This event releases primarily the RelB/p52 dimers, now able to translocate into the nucleus, and together with additional stimulus-specific inputs activate or repress specific genes in cells. Atypical pathways, usual activate NF-κB independent of IKKs, or act on nuclear NF-κB subunits [2].

NF-κB transcription factors are also known to crosstalk to other transcription factors, such as p53, E2F1, and HIF [9,10,11,12], amongst others. HIF is an important family of transcription factors (HIF-1α, HIF-2α, or HIF-3α partnered with HIF-1β), which are known for their role in the response to reduction in oxygen availability or hypoxia [13]. Although HIF is mainly controlled by the activity of the oxygen sensor prolyl-hydroxylases enzymes (PHDs) [14], other mechanisms are also important to control the expression and activity of HIF [10,12,15]. We and others have shown that, in response to canonical pathway activating stimuli, NF-κB is able to induce HIF-1α [16,17,18,19] and HIF-1β [18] directly, and some reports indicate that HIF-3α is also controlled by NF-κB [20,21]. However, so far, no direct control of HIF-2α gene by NF-κB subunits has been reported, although we had previously identified an indirect role for NF-κB to induce HIF-2α in response to TNF-α [18].

TNFSF14 or LIGHT is a non-canonical inducer of NF-κB, analogous to Lymphotoxin-β [22], which also is able to induce the canonical pathway in parallel [23]. It has been shown to control inflammation resolution in the intestine in mice [24], but to also enhance immune-mediated reduction of metastasis in colon cancer [25]. On the other hand, it increases adipose tissue inflammation [26]. Here, we demonstrate that LIGHT can induce the non-canonical NF-κB pathway in two types of cancer cells. In addition, we show that LIGHT can induce the expression and activity of HIF-1α and HIF-2α. Mechanistically, LIGHT induces the mRNA expression of HIF-2α, but not HIF-1α or HIF-1β. LIGHT-induced p52 is required for the elevation of HIF-2α mRNA and protein. Finally, we identify specific binding sites for p52 at the HIF-2α promoter, indicating a direct control of p52 over the HIF-2α gene.

## 2. Material and Methods

### 2.1. Cell Lines and Growth Conditions

All cells were maintained in Dulbecco’s modified Eagle’s medium (DMEM, Lonza, Slough, UK) supplemented with 10% fetal bovine serum (FBS, Invitrogen/ThermoFisher, Paisley, UK), 1% penicillin-streptomycin (Lonza, Slough, UK), and 1% l-glutamine (Lonza, Slough, UK) at 5% CO_2_ and 37 °C for no more than 30 passages. Cells were routinely tested for mycoplasma contamination using MycoAlert Mycoplasma Detection Kit (Lonza, Slough, UK).

Human cervix carcinoma HeLa, and human lung carcinoma A549 cell lines were obtained from the American Type Culture Collection (ATCC).

HeLa-HRE Luciferase cells were previously created in the lab, by the co-transfection of 3xHRE-luciferase construct (a kind gift from G. Melillo, National Cancer Institute, National Institutes of Health, Bethesda, MD, USA) with a puromycin-resistant construct [27]. 

### 2.2. siRNA Cell Transfection

Small interfering RNA oligonucleotides were purchased from Eurofins Genomics and were used in a final concentration of 27 nm. siRNA transfections in all the cell lines were performed using Interferin (Polyplus, Illkirch, France) according to manufacturer’s instructions. Oligonucleotide sequences used for siRNA knockdown are: control, 5′-CAGUCGCGUUUGCGACUGGTT-3′; p100/p52, 5′-CUACGAGGGACCAGCCAAG-3′; RelB, 5′-AAUUGGAGAUCAUCGACGAGU-3′; HIF-1α, 5′-GCAUAUAUCUAGAAGGUAUTT-3′; HIF-2α, 5′-CAGCAUCUUUGAUAGCAGUTT-3′. 

### 2.3. cDNA Transfection

Empty vector control and pCMV4-NIK-HA (a gift from Shao-Cong Sun (Addgene, Teddinghton, UK; plasmid #27554), [28]) were transfected into HeLa cells using TurboFect (ThermoFisher, Paisley, UK), according to the manufacturer’s instructions. Media was changed 4 h post-transfection and cells were lysed for western blot after 48 h.

### 2.4. Cell Treatments

To stimulate the inflammatory response, human recombinant LIGHT (TNFSF14, Peprotech, London, UK) was dissolved in sterile PBS and was used at a final concentration of 100 ng/mL. TNF-α (Peprotech, London, UK) was dissolved in a similar manner, but used at 10 ng/mL.

### 2.5. Luciferase Assay

2 × 10^5^ cells stably transfected with a luciferase reporter gene (based on the HRE present at the iNOS promoter) were seeded in six-well plates, stimulated with LIGHT for the indicated times, and harvested with 400 µL of Passive Lysis Buffer (Promega, Southampton, UK). Luciferase activity was measured according to manufacturer’s instructions (Luciferase Assay System, Promega, Southampton, UK), and was normalised to protein concentration (Bradford, BioRad, Watford, UK). All of the experiments were performed a minimum of three times before calculating average and standard error of the means.

### 2.6. RNA Extraction and Real Time Quantitative PCR Analysis

PeqGOLD total RNA kit (Peqlab, Bishop’s Waltham, UK) or PureLink RNA Mini Kit (Life Technologies/ThermoFisher, Paisley, UK) were used to extract total RNA from cells according to the manufacturer’s instructions. RNA was converted to cDNA using Quantitect Reverse Transcription Kit (Qiagen, Manchester, UK) or First Strand cDNA Synthesis kit (ThermoFisher, Paisley, UK). For quantitative PCR, Brilliant II Sybr green kit (Stratagene/Agilent, Stockport, UK), and recommended MX3005P 96-well skirted plates were used to analyse samples on the Mx3005P qPCR platform (Stratagene/Agilent, Stockport, UK). Alternatively, PerfeCTa Sybr Green FastMix (Quanta Bio, Bervely, MA, US) with ROX dye added in 1:250 ratio, and recommended Microamp Optical 96-well reaction plates were used to analyse samples on the QuantStudio 6 Flex qPCR platform (Applied Biosystem/ThermoFisher, Paisley, UK). Actin was used as normalising gene. RT-PCR results were analysed by the ∆∆Ct method. The primers used for gene expression analysis by RT-PCR are: Actin, For: 5′-CTGGGAGTGGGTGGAGGC-3′, Rev: 5′-TCAACTGGTCTCAAGTCAGTG-3′. HIF-1α, For: 5′-ATAAAGTCTGCAACATGGAAGGT-3′, Rev: 5′-TTTGATGGGTGAGGAATGGGTT-3′. HIF-1β, For: 5′-CAAGCCCCTTGAGAAGTCAG-3′, Rev: 5′-GAGGGGCTAGGCCACTATTC-3′. HIF-2α, For: 5′-TTTGATGTGGAAACGGATGA-3′, Rev: 5′-GGAACCTGCTCTTGCTGTTC-3′. PHD2, For: 5′-GAAAGCCATGGTTGCTTGTT-3′, Rev: 5′-TGTCCTTCTGGAAAAATTCG-3′. RANTES, For: 5′-GTCGTCTTTGTCACCCGAAAG-3′, Rev: 5’-TCCCGAACCCATTTCTTCTCT-3′.

### 2.7. Protein Lysis

Cells were lysed using 100 µL of whole cell protein lysis buffer (20 mm Tris pH 7.6, 150 mm NaCl, 0.75% NP-40, 5 mm NaF, 500 µm Na_3_VO_4_, and 1 Pierce Protease Inhibitor Mini Tablet (EDTA Free, ThermoFisher, Paisley, UK) per 10 mL of buffer). Upon collection, the cells were kept on ice for 30 min before centrifugation at 16,060× *g* at 4 °C for 15 min. The supernatant was collected and stored at −80 °C. 

### 2.8. Western Blotting

Protein concentration was determined using Bradford (BioRad, Watford, UK) method. 20–30 µg of protein was prepared in 2× SDS loading buffer (100 mm Tris-HCl pH 6.8, 20% glycerol, 4% SDS, 200 mm DTT, and Bromophenol Blue), and incubated for 5–10 min at 105 °C. Western blotting was performed as described in [18]. Briefly, samples were loaded into an SDS-page gel (Tris-HCl poly-acrylamide gel) that was previously prepared and run at 80–120 volts in Running Buffer (25 mm Tris, 0.195 M glycine, and 0.1% SDS). The gel was then transferred in a semi-dry transfer (BioRad, Watford, UK) into a PVDF membrane (Millipore, Feltham, UK) for 1.5–2 h at 15 volts/0.80 mA in Transfer Buffer (50 mm Tris, 40 mm glycine, 0.001% SDS and 10% methanol). Then, the membrane was blocked with 10% Milk in TBS-tween buffer (20 mm Tris pH 7.6, 150 mm NaCl, 0.1% Tween) for 10 min, followed by three 5 min washes with TBS-tween buffer. Membranes were incubated with primary antibodies for 1 h at room temperature or overnight at 4 °C, in accordance with primary antibodies’ manufacturer instructions. Membranes were then washed three times with TBS-tween and then incubated with the appropriate secondary HRP antibody. After washes, membranes were developed using ECL solution (Pierce/ThermoFisher, Paisley, UK). Primary antibodies purchased from Cell signaling (Leiden, Holland) were: HIF-1α-OH Pro564 (#3434), HIF-1β (#5537), HIF-2α (#7096), NDRG1 (#5196), NIK (#4994), RelB (#10544), E2F-1 (#3742), p-IKKα/β (#2681), IκB-α (#4821), p-IκB-α (#9246), p-RelA (#3031). Primary antibodies against HIF-1α (#610958) were from BD Biosciences (Workingham, UK), anti-p100/p52 from Millipore (#05-361, Feltham, UK), anti-PHD2 from Bethyl (#A300-322A, Cambridge, UK), anti-Cyclin D1 from Abcam (ab137875, Cambridge, UK), anti-CITED-2 from R&D System (#MAB5005, Abingdon, UK), anti-RelA from Santa Cruz Biotech (#sc-372, Dallas, TX, US), and anti-β-Actin from Proteintech (#66009-1-1g, Manchester, UK).

### 2.9. ChIP-Sequencing Data Analysis

L1236 p52 ChIP sequencing data [29] was downloaded from the NCBI GEO database (GSE63736). Coverage tracks were generated using the R Bioconductor package, Gviz [30].

### 2.10. Chromatin Immunoprecipitation

Chromatin Immunoprecipitation (ChIP) was performed adapting the protocol described in [31]. Cells were plated and grown to 70–80% confluency on 150 mm plates in 16 mL of appropriate culturing media. When necessary cells were treated with LIGHT for 4 h. Then, proteins and chromatin were cross-linked with 1% formaldehyde at 37 °C for 10 min. To quench the cross-linking, glycine was added to a final concentration of 0.125 M for 5 min at 37 °C. Cells were washed twice with ice-cold PBS, then scraped, and centrifuged at 1000 rpm in a Beckman Coulter’s Allegra X-12 benchtop centrifuge for 5 min. The supernatant was removed and the pellet resuspended in 400 μL of ChIP lysis buffer (1% SDS, 10 mm EDTA, 50 mm Tris-HCl pH 8.1, and 1 Pierce Protease Inhibitor Mini Tablet (EDTA Free, ThermoFisher, Paisley, UK), up to 10 mL of buffer), before being snap-frozen in dry ice and stored at −80 °C. Once thawed on ice, 200 µL aliquots of each sample were transferred into 1.5 mL TPX Polymethylpentene (PMP) tubes (Diagenode, Seraing, Belgium) to improve sonication and shearing efficiency. Samples were sonicated in Bioruptor NGS (Diagenode, Seraing, Belgium) at 4 °C for five cycles of 30 s ON/30 s OFF, at high intensity amplitude. This sonication procedure was repeated four times. Supernatants were recovered by centrifugation at maximum speed in a benchtop centrifuge for 10 min at 4 °C prior storage of 10% of each sample as input. Remaining samples were split into 120 μL aliquots before being diluted 10-fold in ChIP Dilution Buffer (1% Triton X-100, 2 mm EDTA, 150 mm NaCl, 20 mm Tris-HCl pH 8.1). Diluted samples were pre-cleared for 2 h at 4 °C by incubation with 2 μg of sheared salmon sperm DNA and 20 μL of protein G-Sepharose (50% slurry), previously washed in cold PBS. Immunoprecipitation was performed overnight on the remaining samples with 2 µg of anti-p52 (santa cruz, #sc-848) or Normal Rabbit IgG (Sigma, Gillinham, UK, #I5381) as negative control, and 0.1% of BRIJ-35 detergent. The following day, immune complexes were captured by incubation with 30 μL of protein G-Sepharose (50% slurry, previously washed with cold PBS) and 2 μg of salmon sperm DNA for 2.5 h at 4 °C. The immunoprecipitates were washed sequentially for 5 min at 4 °C in 1 mL of cold Wash Buffer 1 (0.1% SDS, 1% Triton X-100, 2 mm EDTA, 20 mm Tris-HCl pH 8.1, 150 mm NaCl), 1 mL of cold Wash Buffer 2 (0.1% SDS, 1% Triton X-100, 2 mm EDTA, 20 mm Tris-HCl pH 8.1, 500 mm NaCl), and 1 mL of cold Wash Buffer 3 (0.25 M LiCl, 1% Nonidet P-40, 1% deoxycholate, 1 mm EDTA, 10 mm Tris-HCl pH 8.1). Next, beads were washed twice with 500 µL of Tris-EDTA (TE) buffer. 

Chromatin reverse cross-linking and DNA elution were performed while using the IPure kit v2 (Diagenode) following the manufacturer’s instructions. Briefly, 10% inputs and beads were resuspended in 90 and 100 µL of Elution Buffer mix, respectively. The elution buffer mix was prepared with 115.4 µL of Buffer A and 4.6 µL of Buffer B per sample. All of the samples were incubated overnight at 65 °C on a thermomixer with continuous shaking at 300 rpm. The following day, supernatants were recovered by centrifugation at 1000 rpm for 1 min in a benchtop centrifuge. 2 µL of carrier, 100 µL of 100% isopropanol, and 10 µL of magnetic beads were added to each input and immunoprecipitate sample, then incubated on a rotating wheel (40 rpm) for 10 min at room temperature. After a quick centrifugation, tubes were placed into a magnetic rack for separation of buffer, then discarded, and beads. Captured beads were gently mixed to 100 µL of Wash Buffer 1 (previously diluted with 100% isopropanol with 1:1 ratio), prior incubation on rotating wheel (40 rpm) for 5 min at room temperature. Following another step of buffer separation, beads were gently mixed to 100 µL of Wash Buffer 2 (previously diluted with 100% isopropanol with 1:1 ratio), and incubated on rotating wheel (40 rpm) for 5 min at room temperature. Finally, DNA elution was performed adding to captured beads 25 µL of Buffer C and incubating tubes on a rotating wheel (40 rpm) for 15 min at room temperature. After separation on the magnetic rack, the first fraction of eluted DNA was transferred into a new storage tube, while captured beads were subjected to a second step of DNA elution by adding other 25 µL of Buffer C, to obtain a final volume of 50 µL of purified DNA. 

3 μL DNA were used for RT-PCR analysis with primers that were specifically designed on promoter regions of interest. Specifically, for HIF-2α -2683 κB site, For: 5′-AAGGTGCGGTGGCTTAT-3′, Rev: 5′-GAACTCCTGGCCTCATGT-3′; for HIF-2α -2534 κB site, For: 5′-GGTGGTGCACATCTGTAGT-3′, Rev: 5′-CAGGTTGGAGCGGAGTG-3′; for HIF-2α -1278 κB site, For: 5′-CCCAACCCTTTCTGTGTA-3′, Rev: 5′-CAGAAGCGATTTGAAGAGAAG-3′. HIF-1α -197 κB site, For: 5′-CTGACCGCCTCCTGATTG-3′, Rev: 5′-GATCCAATGGCGAGCGA-3′.

### 2.11. Motif Analysis for κB Sites at the HIF-2α Gene

Consensus κB motif logo was generated from multiple sequence alignment of the 19 non-redundant NF-κB binding sites predicted at the HIF-2α promoter by ALGGEN PROMO, while using WebLogo online tool. Predicted binding sites were centred and trimmed to 10 bps.

### 2.12. Statistical Analysis

Means, standard deviations, and standard error means were calculated prior to performing Student *t* tests on a minimum of three independent experiments and calculating *p*-values. * = *p* < 0.050; ** = *p* < 0.010; and, *** = *p* < 0.001. This analysis was used for comparing two conditions only. One-way Anova analysis, followed by Dunnett test was used for comparing multiple conditions to a control condition only, or Tukey test for multiple pair-wise condition comparisons.

## 3. Results

**Figure 1:**
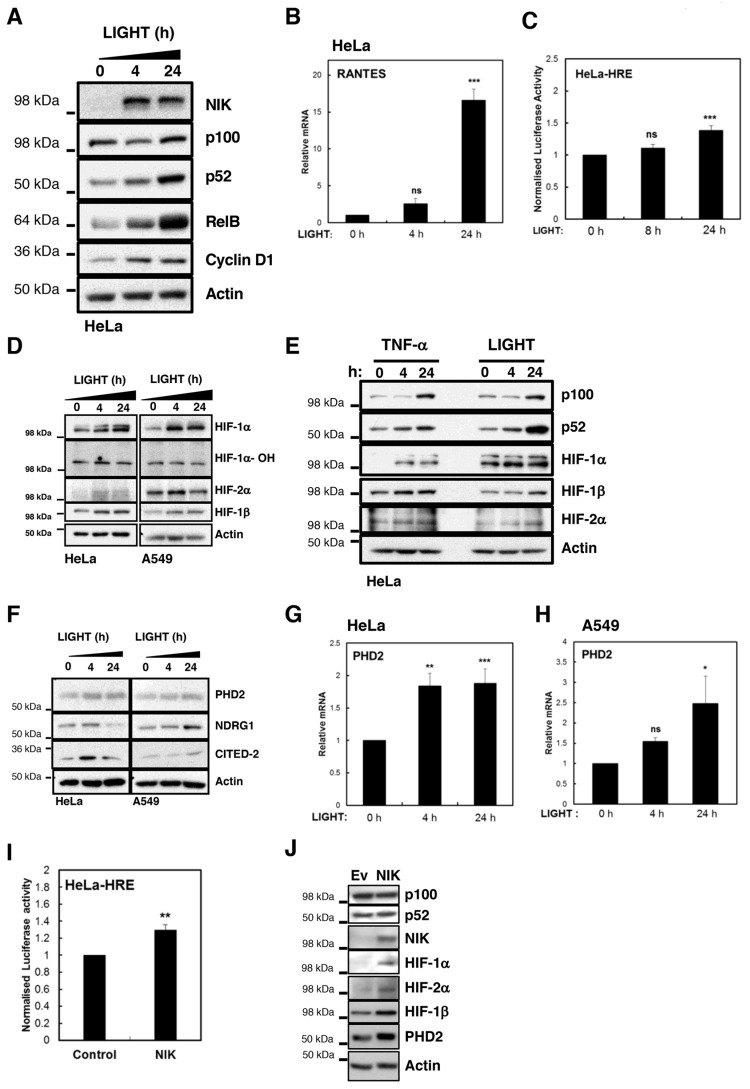
LIGHT, a non-canonical NF-κB inducer, induces HIF expression and activity. (**A**) HeLa cells were treated with 100 ng/mL LIGHT for 0, 4 and 24 h. Whole cell lysates were prepared and analysed by western blot for the indicated non-canonical NF-κB pathway regulators, subunits and target genes. β-Actin was used as loading control. (**B**) HeLa cells were treated with 100 ng/mL LIGHT for 0, 4 and 24 h prior mRNA extraction and RT-qPCR analysis for RANTES transcript, normalised to Actin mRNA levels. All the values were normalised to the untreated sample. The graphs depict mean and SEM determined from at least three independent experiments. One-way Anova analysis was performed and significance determined as follows: ns = not significant, *** *p* ≤ 0.001. (**C**) HeLa cells, stably transfected with HRE luciferase reporter, were treated with 100 ng/mL LIGHT for 8 and 24 h prior to luciferase measurements. All of the values were normalised to the untreated sample. Graph depicts mean and SEM of a minimum of three independent biological experiments. One way Anova analysis was performed and significance determined as follows: ns = not significant, *** *p* ≤ 0.001. (**D**) HeLa and A549 cells were treated with 100 ng/mL LIGHT for 0, 4 and 24 h prior collection of whole cell lysates and western blot analysis for the depicted proteins. β-Actin was used as loading control. (**E**) HeLa cells were treated with 10 ng/mL TNF-α and 100 ng/mL LIGHT for 0, 4 and 24 h. Then, whole cell lysates were collected and western blot analysis was performed for the depicted proteins. β-Actin was used as loading control. (**F**) HeLa and A549 cells were treated with 100 ng/mL LIGHT for 0, 4 and 24 h. Then, whole cell lysates were collected and western blot analysis was performed for a subset of HIF-α specific targets. β-Actin was used as loading control. (**G**) HeLa cells and (**H**) A549 cells were treated with 100 ng/mL LIGHT for 0, 4 and 24 h, prior mRNA extraction and RT-qPCR analysis for PHD2 gene transcript, normalised to Actin mRNA levels. All the values were normalised to the untreated samples. The graph depicts mean and SEM determined from at least three independent biological experiments. One-way Anova analysis was performed and significance determined as follows: ns = not significant, * *p* ≤ 0.05, ** *p* ≤ 0.01, *** *p* ≤ 0.001. (**I**) Analysis of canonical NF-κB signalling following NIK expression vectors for 48 h prior to luciferase measurements. All the values were normalised to the control sample. Graph depicts mean and SEM of a minimum of three independent biological experiments. Student *t*-test analysis was performed and significance determined as follows: ns = not significant, ** *p* ≤ 0.01. (**J**) HeLa cells were transfected with control and NIK expression vectors for 48 h prior to cell lysis. Western blot analysis was performed using the indicating antibodies. β-Actin was used as loading control.

To determine whether LIGHT was able to activate NF-κB in cancer cells, we treated HeLa cells with 100 ng/mL LIGHT for different time periods and analysed markers of non-canonical NF-κB pathway activation (Figure 1A,B). LIGHT treatment readily results in accumulation of NIK, with concomitant increases in the levels of p52 and RelB, as well as one of p52 known targets in cancer cells, Cyclin D1 [31,32]. We could also demonstrate that RANTES (CCL5), which is a known target of non-canonical NF-κB [33], was robustly induced in HeLa and A549 cells following LIGHT treatment (Figure 1B and Figure S1A). Given that there is a possibility of crosstalk between canonical and non-canonical NF-κB activation pathways [34,35], we also analysed markers of canonical NF-κB activation in response to LIGHT treatment (Figure S1B). This analysis revealed that LIGHT is a very weak inducer of canonical activation markers mostly observed following prolonged treatments (Figure S1B).

Given our interest in how NF-κB and HIF crosstalk, we next determined whether LIGHT could induce a HIF transcriptional response in HeLa cells in normoxia (Figure 1C). To this end, we used a generic HIF-dependent gene reporter assay in HeLa cells, treated with LIGHT (Figure 1C). This analysis revealed a small but consistent induction of HIF-dependent luciferase activity upon treatment with LIGHT (Figure 1C). In these cells, reporter activation is dependent on the activity of both HIF-1α and HIF-2α (Figure S2A). To validate this finding, we analysed the levels of HIF proteins in two different cancer cell lines, following treatment with LIGHT (Figure 1D,E). Levels of HIF-1α, HIF-1β, and HIF-2α were induced by LIGHT treatment in both cell lines (Figure 1D). This was not a result of PHD inactivation since we did not detect any reduction in the levels of hydroxylated HIF-1α (Figure 1D).

Interestingly, LIGHT and TNF-α treatment induced HIF levels to a similar degree, suggesting that both cytokines can activate HIF mediated responses in cancer cells (Figure 1E). To investigate this more formally, we investigated the levels of some of HIF targets following LIGHT treatment (Figure 1F–H). These included HIF-1α targets, such as PHD2 (Figure S2B) and HIF-2α genes, such as CITED-2 and NDRG1 (Figure S2C). These results demonstrate the LIGHT is able to activate both NF-κB and HIF transcription factors. 

To determine if activation of the non-canonical NF-κB pathway by another mechanism could lead to similar results, we overexpressed NIK in HeLa cells and investigated the markers of HIF activation (Figure 1I,J). Similar to treatment of cells with LIGHT, NIK overexpression resulted in the activation of HIF-dependent luciferase activity (Figure 1I). Importantly, this was also reflected in the levels of HIF proteins and PHD2 (Figure 1J). Taken together, these results indicate that non-canonical NF-κB activation induces the HIF pathway in cancer cells.

**Figure 2:**
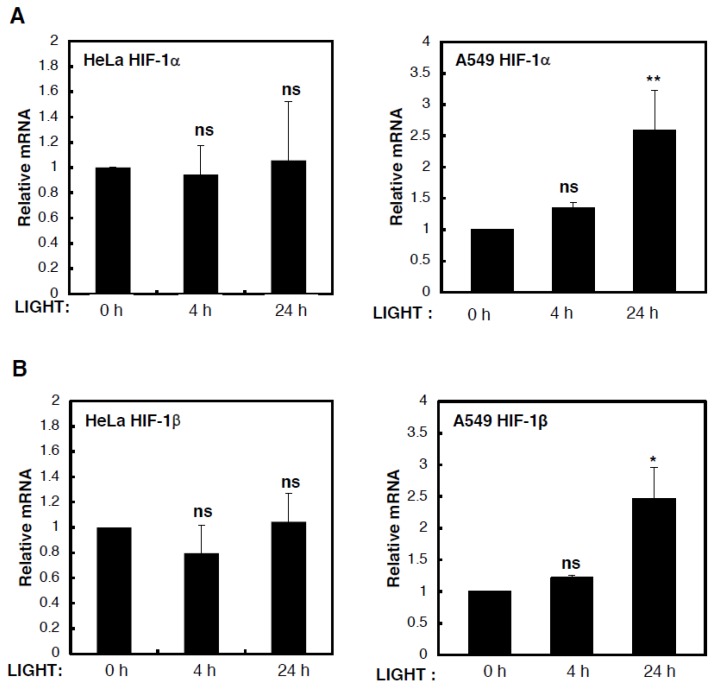

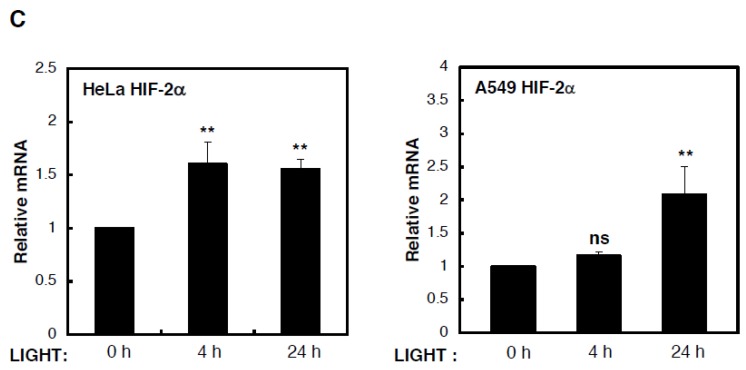
LIGHT induces the expression of HIF-2α mRNA. mRNA was extracted from HeLa and A549 cells, previously treated with 100 ng/mL LIGHT for 0, 4 and 24 h, and RT-qPCR analysis was performed for (**A**) HIF-1α, (**B**) HIF-1β, and (**C**) HIF-2α, normalising to Actin mRNA levels. All the values were normalised to the untreated samples. All graphs indicate mean and SEM determined from at least three independent biological experiments. One way Anova analysis was performed and significance determined as follows: ns = not significant, * *p* ≤ 0.05, ** *p* ≤ 0.01.

To determine the mechanism behind LIGHT-mediated induction of HIF levels and activity, we analysed levels of HIF subunit mRNA in HeLa and A549 cells that were treated with LIGHT for different time periods (Figure 2). Although we could not detect any consistent or statistically significant increases in HIF-1α (Figure 2A) or HIF-1β (Figure 2B) mRNA levels in HeLa cells treated with LIGHT, HIF-2α mRNA levels were induced in a statistically significant manner in such conditions (Figure 2C). On the other hand, in A549 cells, all HIF gene products were induced by LIGHT treatment. This led us to hypothesise, based on our previous experience with cytokine-induced HIF levels [16,18], that in HeLa cells, LIGHT was inducing HIF-2α transcriptionally, while inducing HIF-1β and HIF-1α via an indirect mechanism, much like the one that we previously described for HIF-2α in response to TNF-α [18].

**Figure 3:**
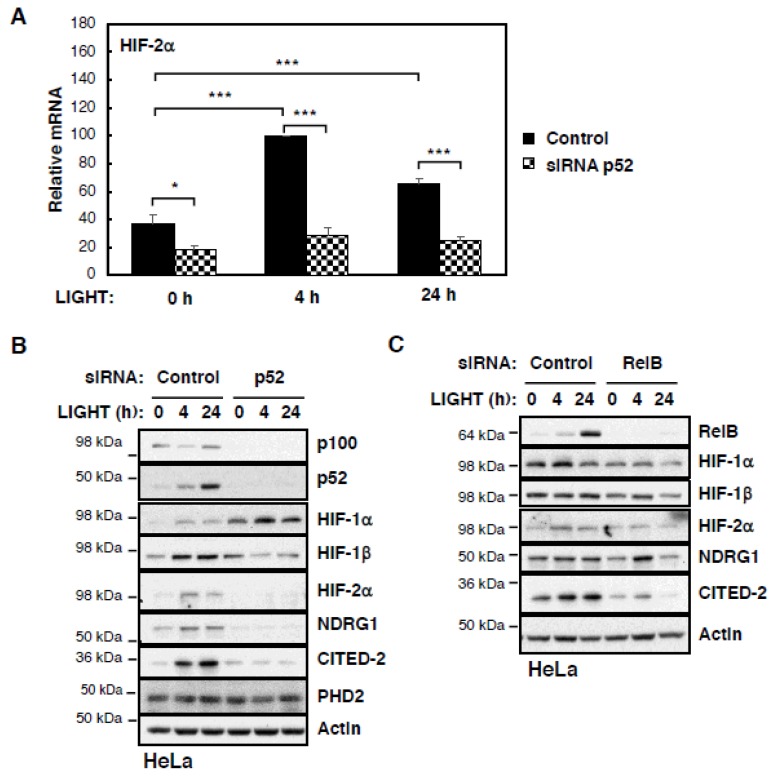
LIGHT-induced HIF-2α expression is p52 dependent. (**A**) HeLa cells were transfected with siRNA control and p52 oligonucleotides and treated with 100 ng/mL LIGHT for the 0, 4, and 24 h prior mRNA extraction. RT-qPCR analysis for HIF-2α gene transcript was performed, normalising to Actin mRNA levels. All of the values were normalised to the control treated with LIGHT for 4 h. The graph shows mean and SEM determined from at least three independent experiments. One-way Anova analysis was performed and significance determined as follows: * *p* ≤ 0.05, *** *p* ≤ 0.001. (**B**,**C**) HeLa cells were transfected with siRNA control and p52 (**B**) or RelB (**C**) oligonucleotides and treated with 100 ng/mL LIGHT for 0, 4, and 24 h. Whole cell lysates were prepared and analysed by western blot to investigate the protein expression of the HIF subunits and a subset of HIF specific targets following treatment. β-Actin was used as loading control.

Given that p52 is strongly induced by LIGHT in our cells, we next investigated whether p52 was contributing to LIGHT-induced HIF levels and activity under conditions of LIGHT treatment. To address this question, we depleted p52 in HeLa cells by siRNA and repeated the treatment with LIGHT (Figure 3A,B). mRNA analysis revealed a strict requirement for p52 in the LIGHT-mediated induction of HIF-2α levels (Figure 3A and Figure S3A). p52 is also required for LIGHT-induced HIF-2α protein and activity in HeLa cells (Figure 3B). Reduced levels of HIF-2α targets CITED-2 and NDRG1 were observed in the absence of p52 despite treatment with LIGHT (Figure 3B). Interestingly, HIF-1β levels were also decreased in the absence of p52, while HIF-1α levels were elevated (Figure 3B). Given that p52 activity is partly dependent on its partner RelB, we extended our analysis to investigate RelB’s contribution to LIGHT-induced HIF levels and activity (Figure 3C). In this case, RelB depletion resulted in reduced levels of all HIF subunits and also the targets that we investigated. This suggests that p52 and RelB have joint functions but also independent functions of each other in the control of different HIF subunits. 

**Figure 4:**
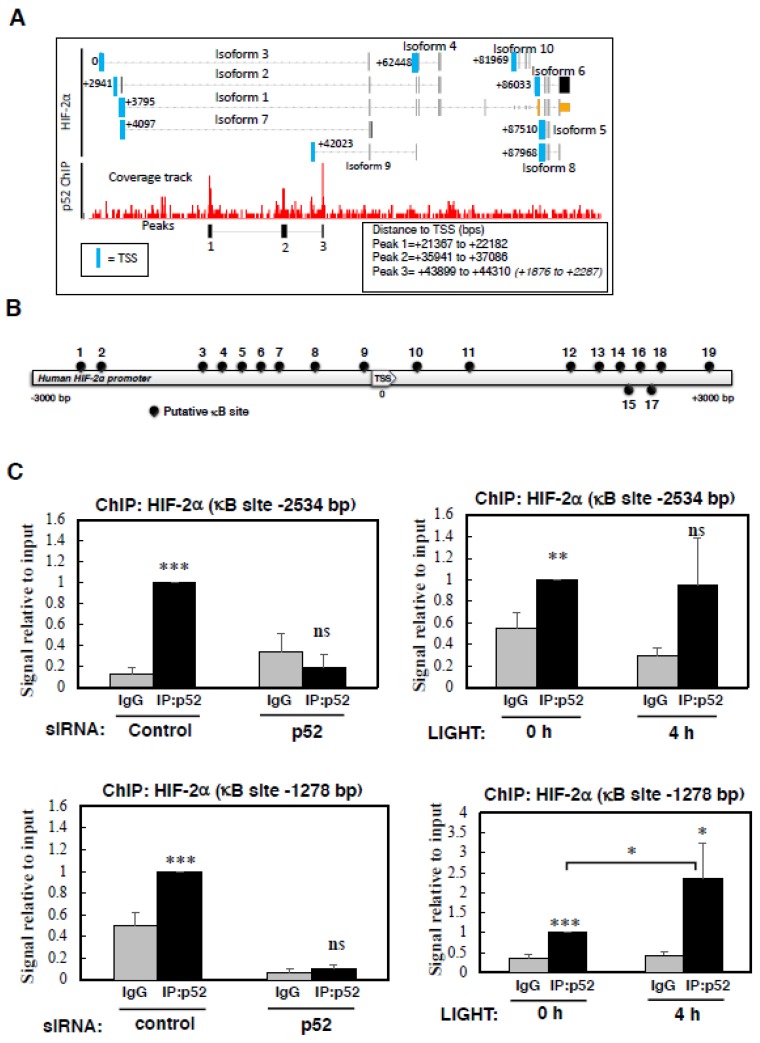
The NF-κB subunit, p52, specifically binds to multiple sites on the HIF-2α promoter. (**A**) Coverage tracks and peaks from L1236 p52 ChIP-seq data at the HIF-2α gene. Peak start and end distances from the HIF-2α transcription start site (TSS) are also shown. *(Numbers in brackets) =* distances from HIF-2α isoform 9 (EPAS-209 (ensembl transcript name)) TSS. (**B**) Schematic diagram showing annotations of putative κB binding sites identified on the promoter region (−/+ 3000 bp from TSS) of HIF-2α gene through bioinformatics analysis conducted using ALGGEN PROMO software. 4 out of 19 sites were subjected to further analysis, to be confirmed as binding sites for the NF-κB subunit p52. (**C**) HeLa cells were transfected with siRNA control and p52 oligonucleotides for 72 h prior to cross-linking and lysis (**left panels**) or treated with 100 ng/mL LIGHT for the 0 and 4 h prior to cross-linking and lysis (**right panels**). Chromatin immunoprecipitations (ChIPs) were performed for the levels of p52 present at the indicated putative κB sites on the HIF-2α promoter. Rabbit IgG was used as antibody control. All of the graphs depict mean and SEM of a minimum of three independent biological experiments. Student *t* test was performed and significance determined as follows: ns = not significant, * *p* ≤ 0.05, **p ≤ 0.01, *** *p* ≤ 0.001. One way Anova analysis was also performed to compare multiple conditions and significance determined as follows: * *p* ≤ 0.05.

In view of the strong effect on HIF-2α mRNA we had observed with p52 depletion, we next investigated whether there was a direct link between these two genes. Initially, we determined if LIGHT had any effect on the levels of E2F1, a transcription factor we had previously showed to control HIF-2α mRNA [36]. This revealed that LIGHT did not alter E2F1 protein levels significantly in the two cell lines that we have analysed (Figure S3B). Due to the availability of an increasing number of publicly available ChIP-sequencing datasets, we interrogated the NCBI GEO database for p52 binding sites at the HIF-2α gene. Despite the limited availability for p52 ChIP-sequencing results, we were able to determine that in lymphoma cells, p52 has been shown to bind to multiple sites in the HIF-2α gene, however there is no documented binding of RelB to the HIF-2α gene [29]. The p52 binding sites are downstream of the HIF-2α promoter, and whilst one binding site is located near the transcription start site (TSS) of isoform 9 (EPAS1-209 (ensembl transcript name)), this isoform does not result in a protein product (Figure 4A). As such, we used bioinformatics analysis utilising different software tools, which revealed a variety of putative κB binding sites present at the HIF-2α promoter (Figure 4B, Table S1). These motifs were mainly based on canonical NF-κB binding (Figure S4A).

To determine if indeed p52 is able to bind to the HIF-2α promoter, we designed several primers to encompass some of the predicted binding sites. As an additional control, we also included siRNA depletion of p52, to really demonstrate specificity of the assay. Although, all antibodies raised against p52 also recognise p100, DNA binding is expected to be mainly due to p52 since p100 is known to act as an IκB-like molecule and sequester p52/RelB in the cytoplasm [37,38]. Using ChIP, we could see that the analysis of -2683 site on the HIF-2α promoter displayed only minimal binding by p52, but the signal obtained was eliminated following p52 depletion (Figure S4B). Analysis of -2534 site revealed a specific binding of p52 which was not induced upon LIGHT treatment (Figure 4C, top panels). This was also the case when we analysed p52 binding to the site that we had previously identified in the HIF-1α promoter [16]. Here, although p52 was specifically bound to the HIF-1α promoter, LIGHT treatment led to a decrease in p52 binding (Figure S4C), further suggesting that LIGHT does not alter HIF-1α transcription in this cell line. 

When we analysed the -1278 site, where we can detect a specific signal for p52 binding, following treatment with LIGHT we could observe a significant increase in p52 binding (Figure 4C, lower panels). Taken together, these results show a direct binding of p52 to the HIF-2α promoter, which we have demonstrated is required for HIF-2α mRNA and protein expression following LIGHT stimulation.

## 4. Discussion

In this report, we demonstrate that the HIF-2α gene is responsive to the activation of non-canonical NF-κB subunits, p52 and RelB, when LIGHT is used as a stimulus. Although other HIF subunits have been shown to be directly controlled by NF-κB [16,18], these have been as a consequence of activation of the canonical NF-κB pathways and have not shown any direct effect on HIF-2α. 

Our results demonstrate that non-canonical activation of NF-κB is also able to induce the expression and activity of HIF transcription factors, but in this case, the effect is mediated by p52 on the HIF-2α gene in both cell lines that we tested. This has implications for situations where non-canonical signalling is high, such as inflammatory conditions and pathogenic infections [39], since this would result in a “pseudo-hypoxic” environment, with HIF transcription factors being activated. In fact, studies in patients with *Helicobacter pilori*, a pathogen that can activate non-canonical NF-κB [39] and can lead to gastric cancer [40], have shown increased levels of HIF-2α in patient biopsies [41]. However, these studies did not determine the mechanism behind the increased level of HIF-2α expression, and as such, more work is necessary to determine whether the mechanism we identified in our study is present under such conditions.

Non-canonical NF-κB is important for B cell development and the expression of Germinal Centres [39], however, whether HIF-2α has any involvement in these functions is still under-investigated. Interestingly, a recent study revealed that the HIF system, including VHL and HIF-1α and HIF-2α, are involved in germinal centre functional regulation, in particular, regarding antibody quality [42]. These findings, together with the results presented here suggest a potential functional crosstalk occurring between non-canonical NF-κB signalling and HIF. In addition, it is known that HIF-2α is expressed in B cell lymphoma [36,43]. As such, our findings raise two possibilities, one that HIF-2α contributes to non-canonical NF-κB signalling in normal B cells, or alternatively, HIF-2α is expressed under pathological conditions of non-canonical NF-κB activation, such as those that are present in cancer cells.

Very little is known about HIF-2α gene regulation. Our own studies, trying to address this lack of knowledge, have found that HIF-2α is regulated by E2F1 [36], and more recently by SP1 and the transcriptional repressor complex SIN3A-HDAC, via the action of SINHCAF [44]. Although no direct interaction between E2F1 and p52 has been described so far, both of them are required for cell cycle progression [31,36], and as such are thought to be part of a similar network. We did not find, however, any effect in the levels of E2F1 following LIGHT treatment, further implicating p52 in the regulation of HIF-2α under the conditions that are investigated here. Although we have found direct binding of p52 to the HIF-2α gene, additional work would be required to determine the importance of these binding sites for HIF-2α expression. Reporter assays, including mutational analysis, and eventually, the ablation of these binding sites in the genome of cells using CRISPR-Cas9 technology would be necessary to answer these questions. Furthermore, ChIP-sequencing analysis for p52 and/or RelB would also allow for us to identify additional bona fide binding sites in cancer cells.

Understanding the differential regulation of the HIF genes will help to determine how the same transcription factor can have opposing roles in different tissues. For example, while it is clear that HIF-2α acts as an oncogene in renal cancer [45], it can act as a tumour suppressor in sarcoma [46] and possibly other tissues as well [44]. Investigating alternative regulatory mechanisms to classic oxygen regulation will help to identify mechanisms responsible for tissue specific actions of these transcription factors.

## Supplementary Materials

The following are available online at http://www.mdpi.com/2073-4409/7/8/102/s1. This file contains four supplementary figures and legends. Figure S1: Analysis of canonical NF-κB signalling following LIGHT treatment. Figure S2: Characterisation of HIF-dependent gene expression following LIGHT treatment. Figure S3: Validation of p52 siRNA-mediated knockdown and LIGHT effects on E2F1 expression. Figure S4: HIF-2α κB motif analysis and validation of NF-κB subunit, p52, ChIP specificity at the HIF-2α and HIF-1α promoters. Table S1: Putative κB genomic locations.
