# Supplementary material for: TNFSF14/LIGHT, a Non-Canonical NF-κB Stimulus, Induces the HIF Pathway
Source: Cells. 2018 Aug 8;7(8):102. doi: 10.3390/cells7080102 (PMC6116154; doi:10.3390/cells7080102)
Supplement: Supplementary file 1 [file cells-07-00102-s001.pdf]

Supplementary Figure Legends-D'Ignazio et al.

Sup. Figure S1. A) Analysis of canonical NF-κB signalling following LIGHT treatment. A) A549 cells were treated with 100 ng/mL LIGHT for 0, 4 and 24 hours prior mRNA extraction and RT-qPCR analysis for RANTES transcript, normalised to Actin mRNA levels. All the values were normalised to the untreated sample. The graph depicts mean and SEM determined from at least three independent biological experiments. One way Anova analysis was performed and significance determined as follows: ns = not significant, ** $p \leq 0.01$. **B)** HeLa and A549 cells were treated with 100ng/mL LIGHT for the indicated periods of time prior to cells lysis. Western blot analysis was performed using the indicated antibodies. β-Actin was used as a loading control. Data is representative of a minimum of three independent biological experiments.

Sup. Figure S2. Characterisation of HIF-dependent gene expression following LIGHT treatment. A) HeLa cells, stably transfected with HRE luciferase reporter, were transfected with control and HIF-1α or HIF-2α siRNA oligonucleotides for 48 hours prior to luciferase measurements. Where indicated, Hx, cells were also exposed to 24 hours of 1% O_2. All the values were normalised to the control hypoxia treated sample. Graph depicts mean and SEM of a minimum of three independent biological experiments. One way Anova analysis was performed and significance determined as follows: *** $p \leq 0.001$. HeLa cells were transfected with control and HIF-1α **B)** or HIF-2α **C)** siRNA oligonucleotides and treated with 100 ng/mL LIGHT for the 0, 4, and 24 hours prior to cell lysis. Whole cell lysates were analysed by western blot using the antibodies depicted. β-Actin was used as loading control. Data is representative of a minimum of three independent biological experiments.

Sup. Figure S3 Validation of p52 siRNA-mediated knockdown and LIGHT effects on E2F1 expression. A) HeLa cells were transfected with siRNA control and p52 oligonucleotides and treated with 100 ng/mL LIGHT for the 0, 4, and 24 hours prior mRNA extraction. RT-qPCR analysis for p52 (NFKB2) gene transcript was performed, normalising to Actin mRNA levels. All the values were normalised to the control treated with LIGHT for 4 hours. The graph shows mean and SEM determined from at least three independent biological experiments. One way Anova analysis was performed and significance determined as follows:

ns = not significant, *** $p \leq 0.001$. **B)** HeLa and A549 were treated with 100 ng/mL LIGHT for the 0, 4, and 24 hours prior to cell lysis. Whole cell lysates were analysed by western blot using the antibodies depicted. β-Actin was used as loading control. Data is representative of a minimum of three independent biological experiments.

Sup. Figure S4. HIF-2α κB motif analysis and validation of NF-κB subunit, p52, ChIP specificity at the HIF-2α and HIF-1α promoters. A) Motif analysis of κB binding sites predicted to be present at the HIF-2α gene. **B)** HeLa cells were transfected with siRNA control and p52 oligonucleotides for 72 hours prior to cross-linking and lysis. Chromatin immunoprecipitations (ChIPs) were performed for the levels of p52 present at the indicated putative κB site on the HIF-2α promoter. Rabbit IgG was used as antibody control. The graph depicts mean and SEM of a minimum of three independent biological experiments. Student t test was performed and significance determined as follows: ns = not significant, *** $p \leq 0.001$. **C**) HeLa cells were transfected with siRNA control and p52 oligonucleotides for 72 hours prior to cross-linking and lysis (left panel) or treated with 100 ng/mL LIGHT for the 0 and 4 hours prior to cross-linking and lysis (right panel). Chromatin immunoprecipitations (ChIPs) were performed for the levels of p52 present at the indicated κB site on the HIF-1α promoter. Rabbit IgG was used as antibody control. The graph depicts mean and SEM of a minimum of three independent experiments. Student t test was performed and significance determined as follows: ns = not significant, *** $p \leq 0.001$.

Sup. Table S1. Putative κB site genomic locations. Site location based on bioinformatic analysis of the HIF-2α promoter.

Figure S1

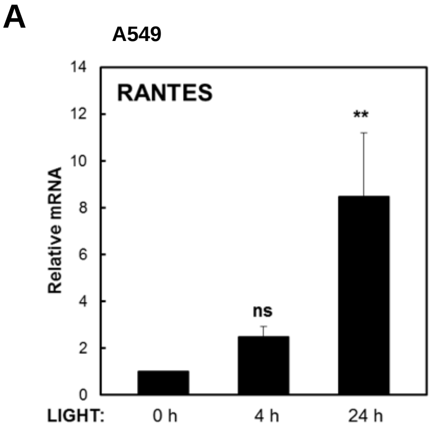
A
A549
RANTES
Relative mRNA
14
12
10
8
6
4
2
0
**
ns
LIGHT:
0 h
4 h
24 h

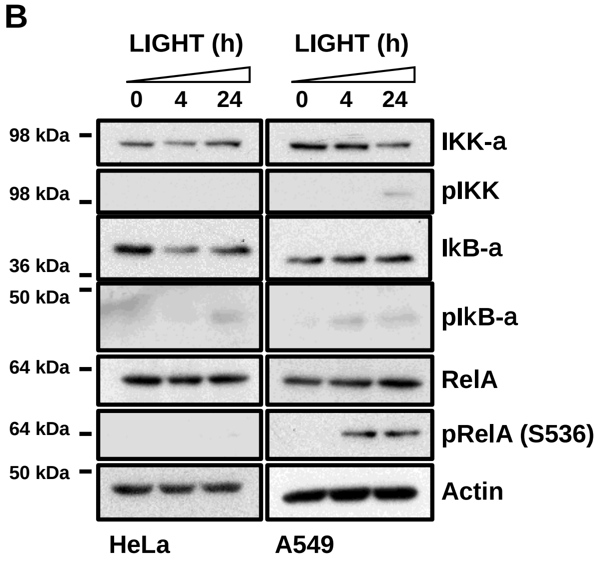
B
LIGHT (h)
LIGHT (h)
0 4 24
0 4 24
98 kDa
98 kDa
36 kDa
50 kDa
64 kDa
64 kDa
50 kDa
IKK-a
pIKK
IkB-a
pIkB-a
RelA
pRelA (S536)
Actin
HeLa
A549

Figure S2

A

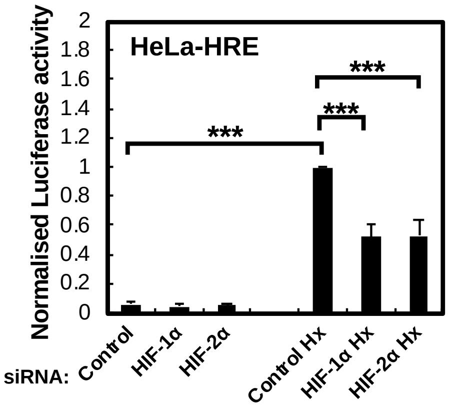

B

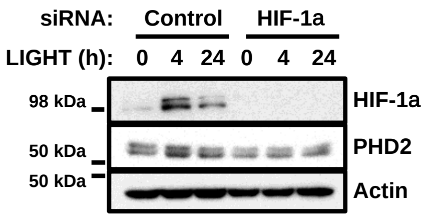

C

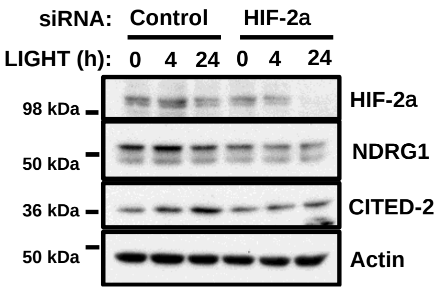

Figure S3

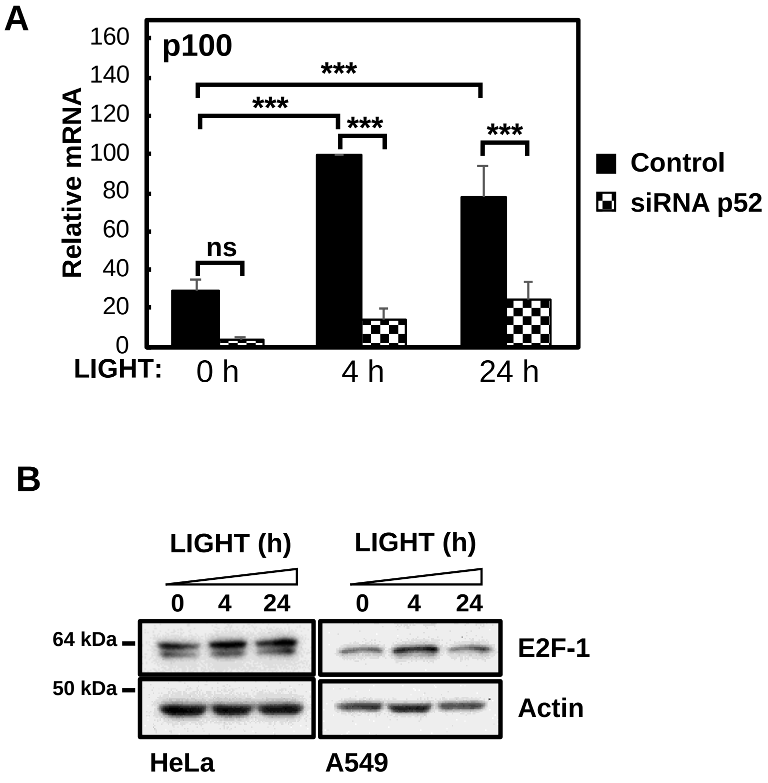
A
p100
Relative mRNA
160
140
120
100
80
60
40
20
0
ns
***
***
***
***
Control
siRNA p52
LIGHT:
0 h
4 h
24 h
B
LIGHT (h)
LIGHT (h)
0
4
24
0
4
24
64 kDa
50 kDa
E2F-1
Actin
HeLa
A549

Figure S4

A

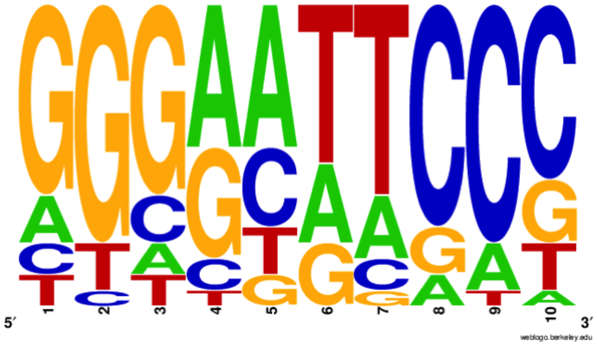

B

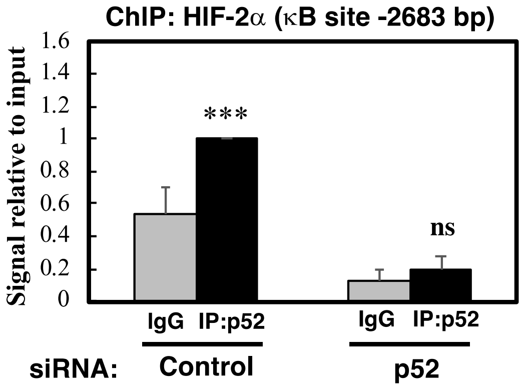

C

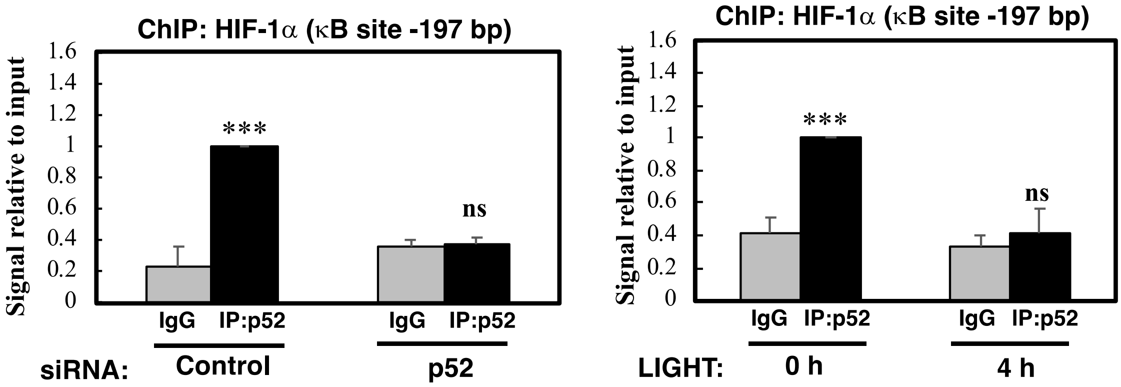

Table S1. Putative kB site genomic locations

kB sites on HIF-2α promoter	Distance from TSS (bp)
**1**	-2683
**2**	-2534
**3**	-1373
**4**	-1278
**5**	-1084
**6**	-1056
**7**	-959
**8**	-887
**9**	-178
**10**	+217
**11**	+751
**12**	+1630
**13**	+1800
**14**	+2008
**15**	+2230
**16**	+2364
**17**	+2451
**18**	+2515
**19**	+2938
